# Social Media Users’ Perceptions of a Wearable Mixed Reality Headset During the COVID-19 Pandemic: Aspect-Based Sentiment Analysis

**DOI:** 10.2196/36850

**Published:** 2022-08-04

**Authors:** Heejin Jeong, Allison Bayro, Sai Patipati Umesh, Kaushal Mamgain, Moontae Lee

**Affiliations:** 1 Department of Mechanical and Industrial Engineering University of Illinois Chicago Chicago, IL United States; 2 Richard and Loan Hill Department of Biomedical Engineering University of Illinois Chicago Chicago, IL United States; 3 Department of Computer Science University of Illinois Chicago Chicago, IL United States; 4 Department of Information and Decision Sciences University of Illinois Chicago Chicago, IL United States

**Keywords:** HoloLens 2, sentiment analysis, natural language processing, Twitter, COVID-19, usability evaluation

## Abstract

**Background:**

Mixed reality (MR) devices provide real-time environments for physical-digital interactions across many domains. Owing to the unprecedented COVID-19 pandemic, MR technologies have supported many new use cases in the health care industry, enabling social distancing practices to minimize the risk of contact and transmission. Despite their novelty and increasing popularity, public evaluations are sparse and often rely on social interactions among users, developers, researchers, and potential buyers.

**Objective:**

The purpose of this study is to use aspect-based sentiment analysis to explore changes in sentiment during the onset of the COVID-19 pandemic as new use cases emerged in the health care industry; to characterize net insights for MR developers, researchers, and users; and to analyze the features of HoloLens 2 (Microsoft Corporation) that are helpful for certain fields and purposes.

**Methods:**

To investigate the user sentiment, we collected 8492 tweets on a wearable MR headset, HoloLens 2, during the initial 10 months since its release in late 2019, coinciding with the onset of the pandemic. Human annotators rated the individual tweets as positive, negative, neutral, or inconclusive. Furthermore, by hiring an interannotator to ensure agreements between the annotators, we used various word vector representations to measure the impact of specific words on sentiment ratings. Following the sentiment classification for each tweet, we trained a model for sentiment analysis via supervised learning.

**Results:**

The results of our sentiment analysis showed that the bag-of-words tokenizing method using a random forest supervised learning approach produced the highest accuracy of the test set at 81.29%. Furthermore, the results showed an apparent change in sentiment during the COVID-19 pandemic period. During the onset of the pandemic, consumer goods were severely affected, which aligns with a drop in both positive and negative sentiment. Following this, there is a sudden spike in positive sentiment, hypothesized to be caused by the new use cases of the device in health care education and training. This pandemic also aligns with drastic changes in the increased number of practical insights for MR developers, researchers, and users and positive net sentiments toward the HoloLens 2 characteristics.

**Conclusions:**

Our approach suggests a simple yet effective way to survey public opinion about new hardware devices quickly. The findings of this study contribute to a holistic understanding of public perception and acceptance of MR technologies during the COVID-19 pandemic and highlight several new implementations of HoloLens 2 in health care. We hope that these findings will inspire new use cases and technological features.

## Introduction

### Background

The release of new virtual reality (VR), augmented reality (AR), or mixed reality (MR) devices elicits a global conversation between VR, AR, and MR developers and users through social media. Such public views may significantly influence the future purchases of potential customers including users, developers, and researchers. Thus, it is essential and meaningful to investigate these views about their usage. This was especially crucial during the unprecedented COVID-19 pandemic, when MR technologies enabled socially distanced education and training in the health care industry. Furthermore, such viewpoints inspire new use cases, which influence health care policy interventions. This investigation offers insights into potential application areas, strengths and weaknesses, and product improvements for future releases. These insights derived from consumer perceptions serve as feedback for the curators to experiment and enhance product capabilities and expand on new use cases inspired by the pandemic.

Previous studies have evaluated the usability and sentiment of VR, AR, and MR headsets [1-3], but there are some limitations. First, there is a lack of evaluations that analyze the usability of sentiments for developers, researchers, and users separately [4]. Moreover, most studies have been evaluated with a limited number of people invited to the laboratory [2,5,6]. Finally, the real-time opinions worldwide have not been reflected [4]. In this study, we propose aspect-based sentiment analysis using Twitter-derived tweets to complement the shortcomings of the existing usability evaluations.

The focus of this study was to explore the usability and sentiment of 1 representative MR headset, Microsoft HoloLens 2, launched in November 2019. HoloLens 2 is the successor product of the initial version released in March 2016. A summary of the comparison between the 2 versions of the HoloLens devices is shown in Table 1. HoloLens 2 has some significant developments compared with the first model. These added developments and features contribute to overall user sentiment. It has new eye-tracking features and gestures. Furthermore, it also has better depth detection, better memory storage, a modern Bluetooth connection, an improved USB port, and a more powerful RAM. Eye tracking enables developers to measure the point of gaze, which benefits eye gaze–based interactions. Kościesza [7] reported that the gesture sensors can recognize up to 25 points of articulation from the fingers and wrist enabling refined object manipulation. In addition, HoloLens 2 also offers a better resolution and field of view. This allows the users to see more without having to turn their heads. Ergonomically, the device also has a knob to enable resizing capabilities for the best fit. A small change in weight makes it slightly more comfortable to wear for a longer duration. The visor flips up, allowing users to wear glasses inside if needed. Thus, HoloLens 2 specifications enable users to manipulate holograms easily and can be used by people of all skill levels for various applications.

**Table 1:** Comparison of HoloLens 1 and 2 (adapted from Kościesza [7] and recreated).

Specification	HoloLens 1	HoloLens 2
Display resolution	1280×720 pixels (per eye)	2048×1080 pixels (per eye)
Field of view	34°	52°
Weight	579 g	566 g
Camera	2.4 MP, HD video	8 MP stills, 1080p video
Audio	Built-in speakers; 3.5-mm jack	Built-in spatial sound; 3.5-mm jack
Built-in microphone	4-microphone array	5-microphone array
Voice command	Yes	Yes
Eye tracking	No	Yes
Biometric security	No	Yes
Hand tracking	1 hand	2 hands full tracking
Price	US $3000	US $3500 or US $99-125 per month
Gestures: press, grab, direct manipulation, touch interaction, scroll with a wave	No	Yes
Memory and storage	1 GB, 64 GB	4 GB, 64 GB

In this study, we analyzed tweets extracted from November 2019 to August 2020, for the first 10 months after the release of HoloLens 2, coinciding with the onset of the pandemic. The opinions about HoloLens 2 shared on Twitter were classified based on (1) positive or negative indicators that evaluate the usability and sensibility of the MR headset (ie, usability, field of view, motion sickness, comfort, immersion, cost, and development) and (2) whether it is an opinion that gives insight to MR developers, researchers, and users (yes or no).

This study has 4 main contributions. First, through aspect-based sentiment analysis, it was possible to denote which feature of HoloLens 2 is helpful for certain fields and purposes. Second, the proposed usability evaluation may be used to develop new VR, AR, and MR devices. Third, it enables rapid analyses using real-time data extracted worldwide. Finally, it facilitates an analysis of sentiment changes over time, as the use cases of the HoloLens2, especially in health care, expanded with the pandemic.

### Previous Work

#### Usability Evaluation Cases of VR, AR, and MR Devices

VR, AR, and MR devices have gained popularity, and therefore, there is much research regarding the use cases of such devices [4,8]. VR is a fully immersive technology that shuts out the real world and transposes users to a web- or internet-based space [9]. In contrast, AR is defined as a real-time view of the physical world enhanced by adding virtual computer–generated information [10]. Finally, MR blends the physical world features of AR and virtual world features of VR to produce an environment in which real and digital objects coexist and interact [9]. Egliston and Carter [11] investigated the relatability of Oculus, a VR product by Facebook, to the lives and values of individuals. Specifically, the researchers used YouTube comments posted on promotional videos for the Oculus. Yildirim et al [12] compared three different gaming platforms to evaluate the effect of VR on the video game user experience: (1) desktop computer, (2) Oculus Rift, and (3) HTC Vive. The applications of such devices are not limited to the gaming field. For example, Bayro et al [13] evaluated the use of VR head-mounted display-based and computer-based remote collaboration solutions. Wei et al [14] assessed the suitability of Google Glass in surgical settings. A substantial amount of the literature gathered between January 2013 and May 2017 suggested a moderate to high acceptability of incorporating Google Glass within various surgical environments. It is also essential to evaluate the customer base of VR, AR, and MR products to understand the real-world applications of such devices. Rauschnabel et al [15] aimed to see what users’ personality traits enable increased willingness to adopt VR technology. The researchers found that consumers who are notably open and emotionally stable are more aware of Google Glass. Furthermore, consumers who recognize the high functional benefits and social conformity of wearables, such as Google Glass, increase technology adoption. A recent study by Ghasemi and Jeong [16] introduced model-based and large-scale video-based remote evaluation tools that could be used to assess the usability of multimodal interaction modalities in MR.

#### Usability Evaluation Cases of HoloLens 1 and 2

Since the launch of HoloLens 1 and HoloLens 2, research has suggested some good use cases across domains. Hammady et al [17] studied how HoloLens provides a good experience when used in museums. This study highlighted the restricted field of view in HoloLens and offered an innovative methodology to improve the accessibility of the spatial UI system, thus resulting in a positive user experience. Hoover et al [18] evaluated the effects of different hardware for providing instructions during complex assembly tasks. The researchers noted that HoloLens users usually have lower error rates than non-AR users [18]. Xue et al [19] investigated user satisfaction in terms of both interaction and enjoyment with the HoloLens device. A total of 142 participants from 3 industrial sectors, including aeronautics, medicine, and astronautics. The researchers concluded that general computer knowledge positively affects user satisfaction despite unfamiliarity with the HoloLens smart glasses. Bräuer and Mazarakis [20] tested the use of HoloLens to increase motivation in AR order-picking tasks through gamification. The researchers found that the participants found the AR application intuitive and satisfying. Levy et al [21] discovered that HoloLens 2 is more efficient than HoloLens 1. Park et al [22] stated that using HoloLens 2 resulted in reduced variability and elevated the performance of all operators performing CT-guided interventions, positively affecting this sector of the health care industry. Furthermore, Thees et al [23] explored the impact of HoloLens 1 on fostering learning and reducing extraneous cognitive processing. This study showed a significantly lower extraneous cognitive load during a physics laboratory experiment using the HoloLens 1.

#### Cases of Sentiment Analysis Based on Social Media

Recently, many studies have used Twitter data to perform sentiment analyses [24]. Carvalho and Plastino [25] highlighted the challenge of this analysis because of the short and informal nature of tweets. Guo et al [26] proposed a Twitter sentiment score model, which exhibits a strong prediction accuracy and reduces the computational burden without the knowledge of historical data. The results of this study provided an efficient model of financial market prediction with an accuracy of 97.87%. Chamlertwat et al [27] proposed a microblog sentiment analysis system that automatically analyzes customer opinions derived from the Twitter microblog service. In the past decade, the Internet of Things (IoT) has also gained popularity. Bian et al [28] mined Twitter to evaluate the public opinion of IoT. Specifically, the researchers collected perceptions of the IoT from multiple Twitter data sources and validated these perceptions against Google Trends. Following this, sentiment analysis was performed to gain insights into public opinion toward the IoT. Mittal and Goel [29] examined the causal relationship between public and market sentiments using a large scale of tweets and a stock market index, the Dow Jones values, from June 2009 to December 2009. Venugopalan and Gupta [30] explored tweet-specific features using domain-independent and domain-specific lexicons to analyze consumer sentiment. In addition, Troisi et al [31] performed a sentiment analysis using data from several social media platforms, including Twitter, to evaluate factors that influence university choice. The researchers noted that the main variable motivating such decision was the training offered, followed closely by physical structure, work opportunities, prestige, and affordability. Nanath and Joy [32] explored the factors that affect COVID-19 pandemic–related content sharing on Twitter by performing natural language processing techniques such as emotion and sentiment analyses. The findings showed that tweets with named entities, expression of negative emotions, referenced mental health, optimistic content, and longer length were more likely shared. Nguyen et al [33] evaluated the association between publicly expressed sentiment toward minorities and resulting birth outcomes. Using Twitter’s streaming application programming interface, the collected and analyzed tweets showed that mothers living in states with the lowest positive sentiment toward minorities had the highest prevalence of low birth weights. Gaspar et al [34] used sentiment analysis techniques to examine affective expressions toward the food contamination caused by enterohemorrhagic *Escherichia coli* in Germany in 2011. The findings highlighted diverse attitudes (positive and negative) and perceived outlooks (threat or challenge), thus emphasizing the ability of sentiment analyses to function as a technique for human-based assessment of stressful events.

Although many studies use data sets of several hundred thousand to millions for sentiment analysis, other researchers report significant findings using <10,000 data points. Myslin et al [35] collected 7362 tobacco-related tweets to develop content and sentiment analysis toward tobacco. The findings suggest that the sentiment toward tobacco was more positive than negative, likely resulting from social image, personal experience, and popular tobacco products. Furthermore, Greaves et al [36] used sentiment analysis techniques to categorize 6412 web-based hospital posts as a positive or negative evaluation of their health care. Using machine learning, the researchers observed moderate associations between predictions on whether patients would recommend a hospital and their responses. More recently, Berkovic et al [37] analyzed 149 arthritis-related tweets to identify topics important to individuals with arthritis during the pandemic and explore the sentiment of such tweets. The results revealed several emerging themes including health care experiences, personal stories, links to relevant blogs, discussion of symptoms, advice sharing, positive messages, and stay-at-home messaging. In addition, the sentiment analysis should address negative concerns about medication shortages, symptom burdens, and the desire for reliable information.

There have also been several sentiment analysis studies in the AR and VR domains. For example, Shahzad et al [38] studied user feedback to evaluate the perception of Fitbit Alta HR (Fitbit). The researchers found that most users spoke highly about such a device. El-Gayar et al [39] used social media analysis techniques to analyze and categorize tweets related to major manufacturers of consumer wearable devices. The analysis provided insight into user priorities related to device characteristics, integration, and wearability issues.

### Benefits of Wearable MR Technologies in Health Care

With the rapid onset of the COVID-19 pandemic, MR technologies have become a revolutionary tool in the health care industry to support educational endeavors, patient care, and rehabilitation. Martin et al [40] explored the capabilities of MR technology to enable telemedicine to support patient care during the pandemic. This study found that the HoloLens2 facilitated a 51.5% reduction in health care workers (HCWs) time exposure to patients with COVID-19 and an 83.1% reduction in the amount of personal protective equipment (PPE) used. This presents a highly beneficial use of MR technology to minimize exposure and optimize PPE use for HCWs. Furthermore, Liu et al [41] evaluated the use of MR techniques to improve medical education and understanding of pulmonary lesions resulting from COVID-19 infection. The researchers concluded that the group’s mean task score using 3D holograms provided by MR techniques was significantly higher than that of the group using standard 2D computed tomography imaging. Moreover, the group using MR technology scored substantially lower for the mental, temporal performance, and frustration subscales on the National Aeronautics and Space Administration Task Load Index questionnaire. These results highlight the use of MR tools in medical education to improve understandability, spatial awareness, and interest and lower the learning curve. Similarly, Muangpoon et al [42] used MR to support benchtop models for digital rectal examinations to improve visualization and learning. The evaluation of such a MR system showed that the increased visualization allowed for enhanced learning, teaching, and assessment of digital rectal examinations. Hilt et al [43] examined the use of MR technologies to provide patient education on myocardial infarction. The researchers concluded that MR technologies act as a practical tool to unite disease perspectives between patients and professionals as well as optimize knowledge transfer. In addition, House et al [44] investigated the use of an MR tool, *VSI Patient Education*, to provide superior education before epilepsy surgery or stereotactic electrode implantation compared with standard 3D rubber brain models. The results showed that the MR tool provided more comprehensible and imaginable patient education than the rubber brain model. In addition, the patients showed a higher preference for the VSI Patient Education tool, emphasizing the benefits of MR tools as the future for patient education. Overall, the rapid acceleration of MR technologies has supported the accessibility and quality of care while also protecting health care staff [40]. When deploying such technologies, topics such as information security, infection control, user experience, and workflow integration must be considered [40]. Such use cases and related requirements must be incorporated into new policy interventions to ensure maximum impact by MR technologies.

## Methods

### Overview

In this study, text data sets were extracted from Twitter. Three human annotators rated the tweets on a positive, negative, neutral, and inconsistent scale for different factors. We used an interannotator and the mean of the ratings to agree with all the human annotators. The annotated tweets were converted into numerical data using 4 word-embedding models: bag-of-words, term frequency–inverse document frequency, Word2vec, and Doc2Vec. Then, we divided the data set into training and testing with a 4:1 ratio and further divided into training and validation in the ratio of 7:3. Our choice to split the data set into the following ratios was derived from prior work on sentiment analysis evaluation. Specifically, Khagi et al [45] evaluated the performance classification accuracy with a 7:3 ratio with a 5-fold cross-validation. Furthermore, Singh and Kumari [46] used a 4:1 training to testing ratio for sentiment classification. We used a stratified random sampling technique to split these data. Stratified random sampling divides the entire population into homogeneous groups called strata (plural for stratum). Random samples were then selected from each stratum. Finally, we used 4 classification models to classify the sentiment of each tweet.

### Data Extraction and Preprocessing

**Figure 1:**
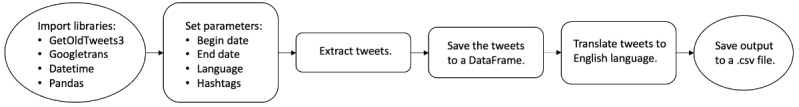
Flowchart of the data extraction process.

The “GetOldTweets3” library from Python was used to extract the tweets. The data corpus consists of tweets posted between November 7, 2019, and August 31, 2020, shortly after the pandemic, which were filtered based on the hashtag, “hololens2,” and relevant terms including “holo lens 2” and “hololens 2.” We downloaded 8492 tweets, which on average consisted of 20 words each. This study also considered tweets in multiple languages. The corpus contained 5379 tweets in English; 2630 tweets in Japanese; 102 tweets in French; and small portions of German, Spanish, Dutch, and Swedish. A translator from the “googletrans” library in Python was used to translate the tweets into English. Googletrans uses the Google Translate Ajax application programming interface to perform these translations. This translation was performed to enable human annotators to rate the sentiment and improve accuracy rather than machine annotators. The data set did not contain retweets, which would add redundancy to the analysis. Quoted tweets were included if additional texts were included in the search term. Figure 1 shows the flowchart of the data extraction process in Jupyter using Python programming language.

After the data collection process, 3 human annotators determined the sentiment of the tweets. Each annotator rated the tweet with respect to the following aspects: usability, field of view, motion sickness, comfort, immersion, cost, and development. This rating was on a scale of positive, negative, neutral, and inconclusive. Positive was rated if the tweet conveyed a positive sentiment toward an attribute. Negative was rated if the tweet conveyed a negative sentiment toward an attribute. Neutral was rated when the tweet did not convey a positive or negative attitude toward an attribute. Finally, inconclusive was rated if the tweet had mixed sentiments or did not have any information related to that specific attribute. Furthermore, human annotators rated the tweets (yes or no) based on the suitability for insights to MR developers, MR user experience researchers, or MR customers and users.

As manually annotating tweets is mostly a subjective process, there were a few instances where the perspective of different annotators was not in agreement. Therefore, to address this challenge, we performed an interannotator agreement. We quantified each positive, negative, neutral, and inconsistent sentiment with a numeric value (ie, 1, −1, 0, and 0). To ease the computation of the interannotator agreement score, the inconsistent label was marked as 0 so that the overall agreement score remained unaffected. The mean of these values was computed using equation (1):

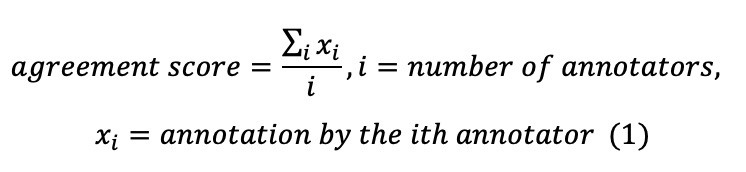



If the mean value was close to −1 and 1, we regarded the annotator perspective as a match. If the mean value was close to 0, we marked that the annotators disagreed with the sentiment conveyed by the tweet. Next, we calculated the average of all the attributes with respect to a tweet to determine the overall sentiment. If this average was positive, we classified the tweet as positive; otherwise, it was classified as negative.

### Word-Embedding Models

#### Bag-of-Words Model

A bag-of-words model represents a method to describe the occurrence of words within a document [47]. It involves two factors: (1) a vocabulary of known words and (2) a measure of the presence of known words. It is referred to as a “bag” of words because the corresponding document is viewed as a set of words rather than a sequence of words. The document’s meaning is often well represented by the set of words, whereas the actual word order is ignored. As such, from the content alone, the document’s meaning can be determined. Zhang et al [48] developed 2 algorithms that do not rely on clustering and achieved competitive performance in object categorization compared with clustering-based bag-of-words representations. They were successful in achieving better results with their approach. Wu et al [49] proposed a bag-of-words model that mapped semantically related features to the same visual words. Their proposed scheme was effective, and it greatly enhanced the performance of the bag-of-words model.

#### Term Frequency–Inverse Document Frequency

The term frequency–inverse document frequency (TF-IDF) is a numerical statistic intended to reflect how important a word is to a document in a collection or corpus [50]. It is one of the most widely used techniques for key word detection [51]. The TF-IDF value increases proportionally with the number of times a word appears in the document. However, it is essential to not only consider the number of times a given word occurs in a document but also consider how frequently the word appears in other documents [51]. For example, certain words, referred to as stopwords, such as “is,” “of,” and “that” frequently appear in documents yet have little importance. To compensate, the TF-IDF value increases with the number of times a word appears in a document but is also offset by the occurrence of that word with a corpus [52]. Peng et al [53] evaluated a novel TF-IDF improved feature weighting approach that reflected the importance of the term among different types of documents. This was achieved by considering the positive or negative set and weighing the term appropriately. This study showed that the term frequency–inverse positive-negative document frequency classifier outperforms the standard TF-IDF technique. In addition, the results of this study highlight the importance of this analysis technique for imbalanced data sets, which, if not accounted for, could lead to erroneous results [54].

#### Word2vec

Word2vec is a combination of models, the continuous bag-of-words and skip-gram, used to represent distributed representations of words in a corpus C [55]. Word2vec is an algorithm that accepts a text corpus as an input and outputs a vector representation for each word [56]. Word2vec outputs word vectors that can be represented as a large piece of text or even the entire article [57]. Unlike most test classification techniques, Word2vec uses both a supervised and unsupervised approach. In particular, it is supervised as the model derives a supervised learning task using continuous bags or words and a skip-gram. Furthermore, it is unsupervised, given that any large corpus of choice can be provided [58]. Word2vec cannot determine the importance of each word within a document; therefore, it is challenging to extract which words hold higher importance, comparatively [58]. Ma et al [59] applied the Word2vec technique in big data processing to cluster similar data and reduce the dimension. The results showed that training data fed into Word2vec decreased the data dimension and sped up multiclass classification. Lilleberg et al [58] found that a combination of Word2vec and TF-IDF outperformed TF-IDF.

#### Doc2Vec

Doc2Vec also uses an unsupervised learning approach to learn document representation [60]. It can be used to identify abnormal comments and recommend relevant topics to users [61,62]. The input of texts (ie, words) per document can be varied, whereas the output is a fixed-length vector [59]. It is a modified version of the Word2vec algorithm using paragraph vectors [63]. Paragraph vectors are unique among all documents, whereas word vectors are shared among all documents. Word vectors can be learned from different documents. Word vectors will be trained during the training phase, while paragraphs will be thrown away after that. During the prediction phase, paragraph vectors will be initialized randomly and computed using word vectors. The main difference between Doc2Vec and Word2Vec is that the latter computes a vector for every word in the document, whereas Doc2Vec computes a vector for the entire document in the corpus. Using Word2Vec and Doc2Vec together will yield significantly better results and promote a thorough study of any document.

### Classification Models

#### Logistic Regression

The logistic regression model is based on the odds of the binary outcomes of interest [64]. For simplicity, one outcome level is designated as the event of interest. In the following text, it is simply called the event. The odds of the event are the ratio of the probability of the event occurring divided by the likelihood of the event not occurring. Odds are often used for gambling, and “even odds” (odds=1) correspond to the event happening half the time. This would be the case for rolling an even number on a single die. The odds for rolling a number <5 would be 2 because rolling a number <5 is twice as likely as rolling a number 5 or 6. Symmetry in the odds is found by taking the reciprocal. The odds of rolling at least a 5 would be 0.5 (=1/2). The logistic regression model takes the natural logarithm of the odds as a regression function of the predictors. With 1 predictor, X, this takes the form ln[odds(Y=1)]=β0+β1X, where ln stands for the natural logarithm, Y is the outcome, where Y=1 occurs when the event occurs and Y=0 when it does not, β0 is the intercept term, and β1 represents the regression coefficient, the change in the logarithm of the event odds with a 1-unit change in the predictor X. The difference in the logarithms of 2 values is equal to the logarithm of the ratio of the 2 values. Thus, by taking the exponential of β1, we obtain the odds ratio corresponding to a 1-unit change in X. The logistic regression model has been used in many social media–based sentiment analysis studies [65-67].

#### Random Forest

Random forest is an ensemble learning method based on the decision tree algorithm [68]. It uses multiple decision trees and merges them to provide absolute and stable outcomes, mostly used for training and class output. Many previous studies successfully used the decision tree and random forest algorithms for sentiment classification of social media data [69-72].

#### XGBoost

The XGBoost (eXtreme Gradient Boos) is a scalable end-to-end tree boosting system for tree boosting, which uses a sparsity aware algorithm to handle sparse data sets [73]. Although the XGBoost uses a representation similar to that of random forest, the prediction error is significantly lower than that of the random forest. Gradient boosting is an approach where new models are created that predict the residuals or errors of prior models, which are then added together to make the final prediction. It is called gradient boosting, as it uses a gradient descent algorithm to minimize the loss when adding new models. The gradient boosting algorithm achieves results faster and performs efficiently compared with other algorithms. Aziz and Dimililer [74] used an ensemble XGBoost classifier to enhance sentiment analysis in social media data and demonstrated an improvement of the sentiment classification performance.

#### Support Vector Machines

A support vector machine (SVM) is a supervised learning model for 2-group classification problems by locating a hyperplane in a multidimensional space that clearly separates the data points [75,76]. The main purpose of SVM is to determine an optimal separating hyperplane that not only separates the data but also ensures that the margin to the data on both sides is as large as possible. First, an optimal solution in a low-dimensional space that can aptly separate the data is evaluated. If this is not possible, the data are mapped to a high-dimensional space by using nonlinear transformation methods. From this, a valid kernel function is selected to determine the optimal linear classification surface. It is highly efficient in separating data into different classes. This allows us to group words into different categories, which helps us access the words easily. The SVM model has been used in various sentiment analysis studies and has produced high classification accuracy [77-79].

### Ethics Approval

This research does not require institutional review board approval because the project does not include any interaction or intervention with human subjects.

## Results

### Model Learning and Performance

Once we determined the classified sentiment for each tweet, we trained a model for sentiment analysis using supervised learning. First, we evaluated the imbalance in the data set: 527 positive tweets and 229 negative tweets. We collected data from 516 unique users in this study. The minimum number of tweets per user was 1, whereas the maximum was 18. The average number of tweets per user was 1.50 (SD 0.3).

To perform supervised learning, it was necessary to preprocess the data. We cleaned the data by removing punctuations, stop words, single characters, and uneven spaces; converting the data to lower case; and stemming on these data. Following preprocessing, we tokenized the data using 4 different techniques: bag-of-words, TF-IDF, Word2vec, and Doc2Vec. Table 2 lists the performance of each model with different word embeddings over a training test ratio of 80:20. This table shows that the bag-of-words tokenizing method using a random forest supervised learning approach produced the highest accuracy of the test set at 81.29%. Furthermore, Textbox 1 summarizes the top words that contribute toward sentiment classification. This textbox highlights various words contributing to sentiments, such as “problem,” “mess,” and “error” for negative and “nice,” “love,” and “achieve” for positive.

**Table 2:** The performance percentage of each model with different work embeddings.

Method and set		Logistic regression	Random forest	XGBoost	SVM^a^
**Bag-of-words**					
	Validation	69.18	72.79	65.40	69.72
	Test	69.03	81.29	72.25	75.48
**TF-IDF^b^**					
	Validation	69.72	74.05	62.16	70.81
	Test	74.83	76.12	74.83	78.70
**Word2vec**					
	Validation	65.40	68.10	68.84	67.02
	Test	72.25	74.83	77.41	71.61
**Doc2Vec**					
	Validation	66.48	67.56	66.48	68.10
	Test	70.32	67.74	70.32	69.03

^a^SVM: support vector machine.

^b^TF-IDF: term frequency–inverse document frequency.

**Most significant words used in the sentiment analysis.**

- mrdevdays
- talking
- thinking
- pc
- mvis
- azure
- mess
- market
- think
- announced
- use
- knowledge
- yotiky
- markets
- lightning
- firefox
- achieve
- babylon
- hatenablog
- nice
- playing
- july
- emulator
- available
- hololens2
- microvision
- love
- today
- general
- keynote
- mxdrealitydev
- hololens
- snapchat
- terrible
- solve
- problem
- forehead
- time
- buy
- msdevirl
- probably
- million
- altspacevr
- microsoft
- half
- nreal
- procedure
- error
- office

### Insights From the Perspective of the COVID-19 Pandemic and Health Care

Following the determination of an appropriate classification model, we evaluated the reasoning for positive or negative tweet classification. Upon investigation, words like “COVID,” “pandemic,” “patients,” and “health care” were all associated with the positive sentiment. Further evaluation showed that the use of HoloLens 2 is highly encouraged in the health care industry in several respects. First, tweets showed the use of HoloLens2 to enable virtual appointments in times of unprecedented crisis. As such, HCW found HoloLens2 to be a vital tool to improve safety and quality of care while also being easy to set up and comfortable to wear. This finding is significant as it supports previous studies evaluating the capabilities of MR technology to permit telemedicine [40]. Other tweets highlighted the use of HoloLens2 to facilitate education and training during the pandemic. Specifically, the HoloLens2 enabled HCW to practice coronavirus identification in a socially distanced manner, which minimized the risk of contact and transmission. Similarly, this finding is significant as it supports prior works relating to the use of MR tools to improve medical education and understanding [41-43]. The following are examples of tweets that qualitatively support these insights:

1. We are revolutionizing healthcare using @Microsoft #HoloLens2 to deliver remote care in #COVID19! Staff found it easy to set up, comfortable to wear, improved quality of care. #Hololens2 is helping keep our #healthcareworkers stay safe on the frontline!
2. “Use of #HoloLens Mixed Reality Headset for Protecting Health Care Workers During the #COVID19 Pandemic”: Prospective study used @Microsoft HoloLens2 to support remote patient care for hospitalized patients. Reduced exposure time by 51% & PPE usage by 83%:
3. Nowadays #medical industry getting lots of advancement with recent tech. There are many notable advantages of #Microsoft #HoloLens that prove that the future of #healthcare is heavily reliant on #MixedReality technology #MR #XR #Hololens2 #AR #Remote
4. #HoloLens2 helps safely train doctors to identify #coronavirus in patients. #MixedReality offers the perfect, socially-distanced or remote training experience, minimizing contact, risk and transmission.
5. Use of the HoloLens2 Mixed Reality Headset for Protecting Health Care Workers During the COVID-19 Pandemic: Prospective, Observational Evaluation

**Figure 2:**
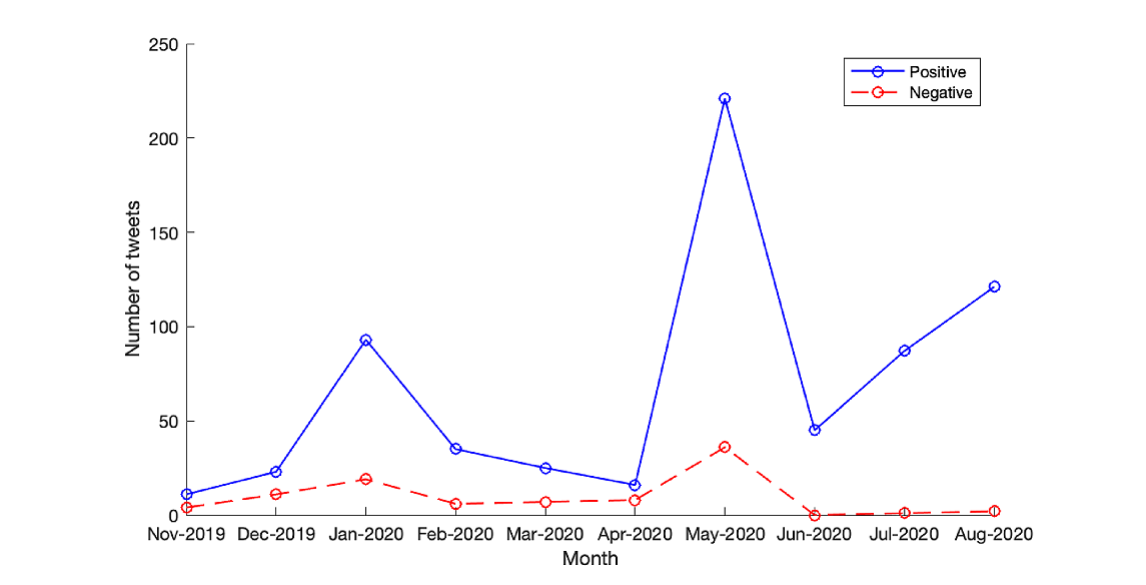
Tweet sentiment over time.

Changes in sentiment toward HoloLens2 throughout the pandemic were also evaluated. In November 2019, when HoloLens 2 was released, there was no significant difference in the positive and negative sentiment (Figure 2). This is likely caused by consumer delay to learn about the product’s arrival in the market supported by the low tweet volumes of both positive and negative sentiments. In December 2019 and January 2020, a significant increase in the positive view was observed, likely caused by consumer interest in the newly released product. In February 2020, the onset of the pandemic occurred, which resulted in the severely affected sales of consumer goods. This period aligns with the drop in general sentiment on both sides. However, the general sentiment of HoloLens 2 seems to be positive despite affected sales. In May 2020, there was a sudden increase in positive sentiment. It is hypothesized that consumers, especially in health care, noticed the device’s benefits to minimize the risk of contraction and transmission. Following this significant change in sentiment, the negative sentiment toward the device almost dropped to 0, highlighting the continued positive role of HoloLens2 during the pandemic.

### Insights for MR Developers, Researchers, and Users

**Figure 3:**
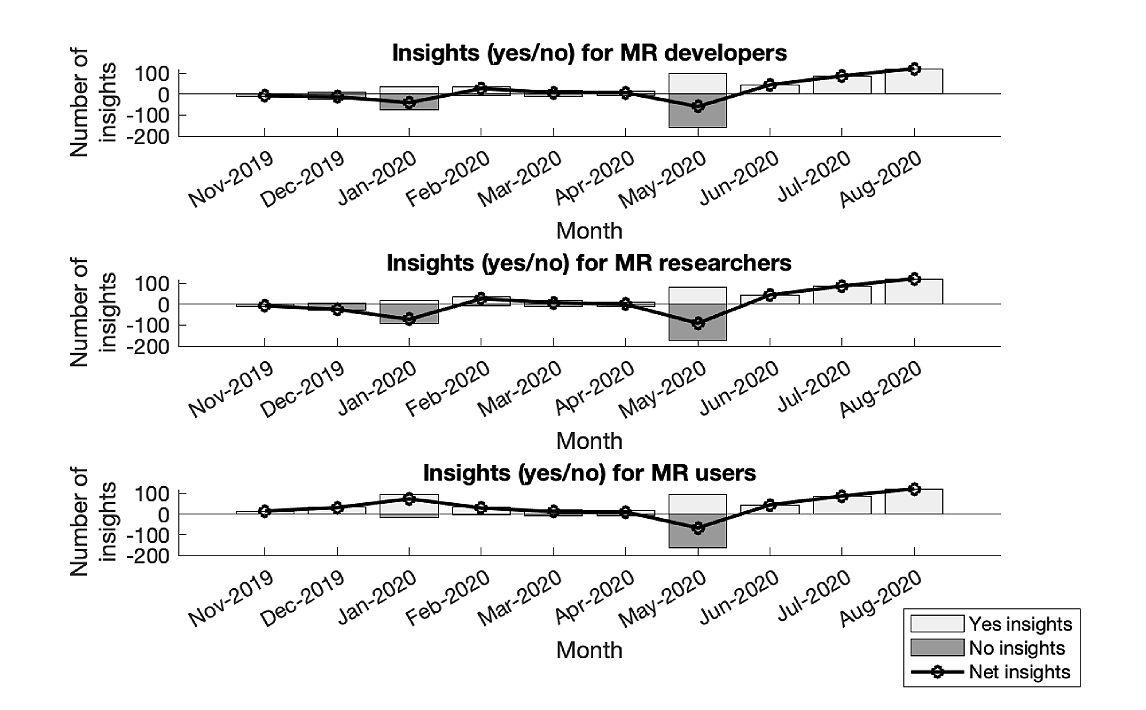
Suitability of tweets to provide insights to mixed reality (MR) developers, researchers, and users after its release.

Figure 3 breaks down the tweets into useful insights for MR developers, defined as individuals developing features of the technology, researchers, defined as individuals using the device for research endeavors (ie, usability analyses), and users, defined as individuals using the device for leisure. The green bars represent tweets classified as suitable insights, and red bars as not suitable. Furthermore, we calculated the net insights, indicated by the black line, as the suitable insights (yes) minus the not suitable insights (no). In the first few months, the data are distributed equally on both sides, and the net insight is approximately 0. In May 2020, there is a drastic difference in the distribution. We presume that this sudden charge is because of the largely changing technology uses caused by the pandemic. Following, we predict that the steady increase in suitable insights results from individuals becoming more acclimated to the technology-driven, remote lifestyle.

### Analysis of HoloLens2 Characteristics

**Figure 4:**
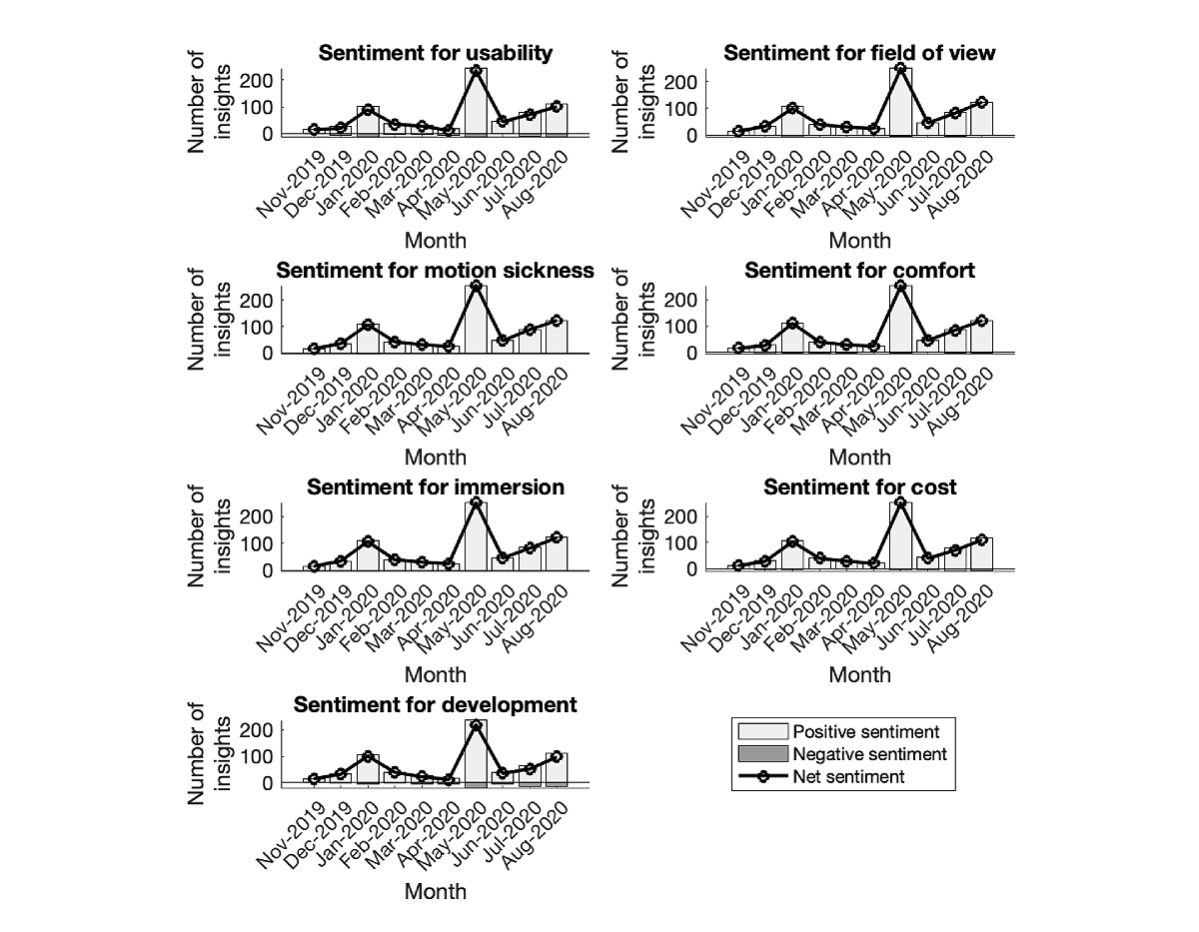
Positive, negative, and net sentiments related to various factors over 10 months.

Table 3 shows the net sentiment of various factors related to HoloLens 2 over the analyzed period. Furthermore, Figure 4 illustrates the number of positive sentiments as green bars, negative sentiments as red bars, and net sentiment as the black line for all factors. We calculated the net sentiment as the number of tweets with positive sentiment minus the number of tweets with negative sentiment. The results show that net sentiment is exclusively positive for all factors in all the months studied. It shows a positive trend in usability, field of view, motion sickness, comfort, immersion, cost, and development. All these factors contributed to positive sentiment toward HoloLens 2. This trend can be credited to the impact of the COVID-19 pandemic as the number of people depending on this device increased.

**Table 3:** Stacked net sentiments related to various factors over 10 months.

Month	Usability	Field of view	Motion sickness	Comfort	Immersion	Cost	Development
November 19	+15	+15	+15	+15	+15	+11	+15
December 19	+20	+32	+34	+26	+34	+28	+32
January 20	+90	+100	+106	+110	+106	+102	+100
February 20	+35	+39	+41	+39	+39	+39	+39
March 20	+28	+30	+30	+28	+30	+28	+22
April 20	+12	+24	+24	+24	+24	+20	+12
May 20	+235	+249	+257	+253	+251	+251	+219
June 20	+45	+45	+45	+45	+45	+39	+35
July 20	+72	+82	+88	+84	+82	+70	+52
August 20	+101	+123	+123	+121	+121	+109	+97

## Discussion

### Principal Findings

The bag-of-words tokenizing method, using a random forest supervised learning approach, provided the highest accuracy of the test set at 81.29%, according to the results of our sentiment analysis. Furthermore, the findings reveal an apparent shift in public opinion during the pandemic. Consumer products were significantly affected during the pandemic’s start, which coincided with a dip in both positive and negative emotion. Following that, there is a sharp increase in positive feeling, which is thought to be because of the device’s new applications in health care teaching and training. This coincides with significant shifts in the number of practical insights for MR developers, researchers, and users, as well as positive net attitudes for HoloLens 2 features.

Twitter is one of the most popular social media platforms worldwide. In this study, tweets related to HoloLens 2 were obtained; however, they did not cover all opinions. We only used tweets with the hashtag “hololens2.” Therefore, many tweets related to this topic, without the hashtag, might have been left out. In addition, this resulted in a relatively small sample size comparatively. Furthermore, some individuals might use other platforms to state their opinion about a particular device. For example, some individuals tend to make reviews or first-opinion videos of devices on platforms such as YouTube, which generate much discussion in the comments. These comments also contribute to consumer perceptions of the product. In addition, we could have explored other social media platforms, such as Instagram and Facebook. The literature supports the use of YouTube, Instagram, and Facebook for sentiment analysis. For example, sentiment analysis has been studied to determine the most relevant and popular video on YouTube according to the search [80]. Furthermore, a deep neural network can be used to propose a sentiment analysis model of YouTube comments [81]. Other researchers used a sentiment analysis tool to measure the proposed social value of each image [82]. Ortigosa et al [83] stated that adaptive e-learning systems could use sentiment analysis to support personalized learning. Adding additional platforms in this study would contribute to a greater understanding of consumer perception. Finally, the extent to which the data were sampled may introduce some biases. Less than half of the adults regularly use Twitter; individuals between the ages of 18 and 29 years as well as minorities are highly represented on Twitter compared with the general population, and Twitter consists of almost entirely passive users (<50 tweets per year) and very active users (>1000 tweets per year) [84]. Therefore, these limitations may have resulted in certain samples of the population being more represented than others.

The onset of the pandemic occurred from February 2020. During the first couple of months, we observed a sudden increase in the popularity of HoloLens 2, which was primarily attributed to new use cases in the health care field. In addition, this change can likely be credited to the large shift to working or studying from home. This analysis covered only a portion of the pandemic when the world began adapting to new routines, technologies, and lifestyles. It would have been beneficial to include tweets made a couple of months after August 2020, as this was the period when people were more adapted to working and studying from home. Including more months would provide increased insight on user sentiment over time through the pandemic, enabling a more thorough understanding.

### Conclusions

In this study, we used aspect-based sentiment analysis to study the usability of HoloLens 2. We extracted data from Twitter based on the hashtag “hololens2” to explore user perception about HoloLens 2. We accumulated 8492 tweets and translated the non-English tweets into English using the “googletrans” library in Python. After the data collection process, human annotators rated the tweets on a positive, negative, neutral, and inconsistent scale for 7 different factors and determined the suitability of the tweets to provide insights for MR developers, researchers, and users. We used an interannotator and rating average to ensure agreement among the human annotators. The results show a clear indication between the positive and negative sentiments toward HoloLens 2. Specifically, we observed that the positive sentiment toward the device grew during the onset of the COVID-19 pandemic, whereas the negative sentiment decreased. By separating the most popular words from both sentiments, we identified the positive and negative aspects of the device. We also observed that HoloLens 2 was highly encouraged in the health care industry. A close evaluation of tweets found that HoloLens 2 enabled virtual appointments, supported medical training, and provided patient education. As such, this thematic analysis showed that HoloLens 2 facilitated social distance practices, which largely minimized the risk of contraction and transmission. The findings of this study contribute to a more holistic understanding of public perception and acceptance of VR and AR technologies, especially during the unprecedented COVID-19 pandemic. Further, these findings highlight several new implementations of HoloLens 2 in health care, which may inspire future use cases. In future work, more data from various social media platforms will be included and compared to improve the effectiveness of this process.

## Abbreviations

AR: augmented reality
HCW: health care worker
IoT: Internet of Things
MR: mixed reality
PPE: personal protective equipment
SVM: support vector machine
TF-IDF: term frequency–inverse document frequency
VR: virtual reality

## References

[1] Martinez-Millana A, Bayo-Monton J, Lizondo A, Fernandez-Llatas C, Traver V (2016). Evaluation of Google glass technical limitations on their integration in medical systems. Sensors (Basel).

[2] Broach J, Hart A, Griswold M, Lai J, Boyer EW, Skolnik AB, Chai PR (2018). Usability and reliability of smart glasses for secondary triage during mass casualty incidents. Proc Annu Hawaii Int Conf Syst Sci.

[3] O'Hagan J, Khamis M, Williamson JR (2021). Proceedings of the International Workshop on Immersive Mixed and Virtual Environment Systems (MMVE '21).

[4] Dey A, Billinghurst M, Lindeman RW, Swan JE (2018). A systematic review of 10 years of augmented reality usability studies: 2005 to 2014. Front Robot AI.

[5] Pereira R, Moore HF, Gheisari M, Esmaeili B (2018). Development and usability testing of a panoramic augmented reality environment for fall hazard safety training. Advances in Informatics and Computing in Civil and Construction Engineering.

[6] Vinci C, Brandon KO, Kleinjan M, Hernandez LM, Sawyer LE, Haneke J, Sutton SK, Brandon TH (2020). Augmented reality for smoking cessation: development and usability study. JMIR Mhealth Uhealth.

[7] HoloLens 2 vs HoloLens 1: what’s new?. 4Experience Virtual Reality Studio.

[8] Geszten D, Komlódi A, Hercegfi K, Hámornik B, Young A, Köles M, Lutters2 WG (2018). A content-analysis approach for exploring usability problems in a collaborative virtual environment. Acta Polytechnica Hungarica.

[9] Carroll WM (2020). Emerging Technologies for Nurses Implications for Practice.

[10] Carmigniani J, Furht B, Anisetti M, Ceravolo P, Damiani E, Ivkovic M (2010). Augmented reality technologies, systems and applications. Multimed Tools Appl.

[11] Egliston B, Carter M (2020). Oculus imaginaries: the promises and perils of Facebook’s virtual reality. New Media Soc.

[12] Yildirim C, Carroll M, Hufnal D, Johnson T, Pericles S (2018). Video game user experience: to VR, or not to VR?. Proceedings of the 2018 IEEE Games, Entertainment, Media Conference (GEM).

[13] Bayro A, Ghasemi Y, Jeong H (2022). Subjective and objective analyses of collaboration and co-presence in a virtual reality remote environment. Proceedings of the 2022 IEEE Conference on Virtual Reality and 3D User Interfaces Abstracts and Workshops (VRW).

[14] Wei NJ, Dougherty B, Myers A, Badawy SM (2018). Using google glass in surgical settings: systematic review. JMIR Mhealth Uhealth.

[15] Rauschnabel PA, Brem A, Ivens BS (2015). Who will buy smart glasses? Empirical results of two pre-market-entry studies on the role of personality in individual awareness and intended adoption of Google Glass wearables. Comput Human Behav.

[16] Ghasemi Y, Jeong H Model-based task analysis and large-scale video-based remote evaluation methods for extended reality research. arXiv..

[17] Hammady R, Ma M, Strathearn C (2019). User experience design for mixed reality: a case study of HoloLens in museum. Int J Technol Market.

[18] Hoover M, Miller J, Gilbert S, Winer E (2020). Measuring the performance impact of using the Microsoft HoloLens 1 to provide guided assembly work instructions. J Comput Inf Sci Eng.

[19] Xue H, Sharma P, Wild F (2019). User satisfaction in augmented reality-based training using Microsoft HoloLens. Computers.

[20] AR in order-picking – experimental evidence with Microsoft HoloLens. Mensch und Computer 2018 - Workshopband.

[21] Levy JB, Kong E, Johnson N, Khetarpal A, Tomlinson J, Martin GF, Tanna A (2021). The mixed reality medical ward round with the MS HoloLens 2: innovation in reducing COVID-19 transmission and PPE usage. Future Healthc J.

[22] Park BJ, Hunt SJ, Nadolski GJ, Gade TP (2020). Augmented reality improves procedural efficiency and reduces radiation dose for CT-guided lesion targeting: a phantom study using HoloLens 2. Sci Rep.

[23] Thees M, Kapp S, Strzys MP, Beil F, Lukowicz P, Kuhn J (2020). Effects of augmented reality on learning and cognitive load in university physics laboratory courses. Comput Human Behav.

[24] Kharde AV, Sonawane S (2016). Sentiment analysis of twitter data: a survey of techniques. Int J Comput Application.

[25] Carvalho J, Plastino A (2020). On the evaluation and combination of state-of-the-art features in Twitter sentiment analysis. Artif Intell Rev.

[26] Guo X, Li J (2019). A novel Twitter sentiment analysis model with baseline correlation for financial market prediction with improved efficiency. Proceedings of the 2019 Sixth International Conference on Social Networks Analysis, Management and Security (SNAMS).

[27] Chamlertwat W, Bhattarakosol P, Rungkasiri T, Haruechaiyasak C (2012). Discovering consumer insight from Twitter via sentiment analysis. J Universal Comput Sci.

[28] Bian J, Yoshigoe K, Hicks A, Yuan J, He Z, Xie M, Guo Y, Prosperi M, Salloum R, Modave F (2016). Mining Twitter to assess the public perception of the "Internet of Things". PLoS One.

[29] Mittal A, Goel A Stock prediction using twitter sentiment analysis. Stanford.

[30] Venugopalan M, Gupta D (2015). Exploring sentiment analysis on twitter data. Proceedings of the 2015 Eighth International Conference on Contemporary Computing (IC3).

[31] Troisi O, Grimaldi M, Loia F, Maione G (2018). Big data and sentiment analysis to highlight decision behaviours: a case study for student population. Behav Inform Technol.

[32] Nanath K, Joy G (2021). Leveraging Twitter data to analyze the virality of Covid-19 tweets: a text mining approach. Behav Inform Technol.

[33] Nguyen TT, Meng H, Sandeep S, McCullough M, Yu W, Lau Y, Huang D, Nguyen QC (2018). Twitter-derived measures of sentiment towards minorities (2015-2016) and associations with low birth weight and preterm birth in the United States. Comput Human Behav.

[34] Gaspar R, Pedro C, Panagiotopoulos P, Seibt B (2016). Beyond positive or negative: qualitative sentiment analysis of social media reactions to unexpected stressful events. Comput Human Behav.

[35] Myslín M, Zhu S, Chapman W, Conway M (2013). Using twitter to examine smoking behavior and perceptions of emerging tobacco products. J Med Internet Res.

[36] Greaves F, Ramirez-Cano D, Millett C, Darzi A, Donaldson L (2013). Use of sentiment analysis for capturing patient experience from free-text comments posted online. J Med Internet Res.

[37] Berkovic D, Ackerman IN, Briggs AM, Ayton D (2020). Tweets by people with arthritis during the COVID-19 pandemic: content and sentiment analysis. J Med Internet Res.

[38] Shahzad K, Malik MK, Mehmood K (2020). Perception of wearable intelligent devices: a case of fitbit-alta-HR. Intelligent Technologies and Applications.

[39] El-Gayar O, Nasralah T, Noshokaty AE (2019). Wearable devices for health and wellbeing: design Insights from Twitter. Proceedings of the 52nd Hawaii International Conference on System Sciences.

[40] Martin G, Koizia L, Kooner A, Cafferkey J, Ross C, Purkayastha S, Sivananthan A, Tanna A, Pratt P, Kinross J, PanSurg Collaborative (2020). Use of the HoloLens2 mixed reality headset for protecting health care workers during the COVID-19 pandemic: prospective, observational evaluation. J Med Internet Res.

[41] Liu S, Xie M, Zhang Z, Wu X, Gao F, Lu L, Zhang J, Xie Y, Yang F, Ye Z (2021). A 3D hologram with mixed reality techniques to improve understanding of pulmonary lesions caused by COVID-19: randomized controlled trial. J Med Internet Res.

[42] Muangpoon T, Haghighi Osgouei R, Escobar-Castillejos D, Kontovounisios C, Bello F (2020). Augmented reality system for digital rectal examination training and assessment: system validation. J Med Internet Res.

[43] Hilt AD, Mamaqi Kapllani K, Hierck BP, Kemp AC, Albayrak A, Melles M, Schalij MJ, Scherptong RW (2020). Perspectives of patients and professionals on information and education after myocardial infarction with insight for mixed reality implementation: cross-sectional interview study. JMIR Hum Factors.

[44] House PM, Pelzl S, Furrer S, Lanz M, Simova O, Voges B, Stodieck SR, Brückner KE (2020). Use of the mixed reality tool "VSI Patient Education" for more comprehensible and imaginable patient educations before epilepsy surgery and stereotactic implantation of DBS or stereo-EEG electrodes. Epilepsy Res.

[45] Khagi B, Kwon G, Lama R (2019). Comparative analysis of Alzheimer's disease classification by CDR level using CNN, feature selection, and machine‐learning techniques. Int J Imaging Syst Technol.

[46] Singh T, Kumari M (2016). Role of text pre-processing in twitter sentiment analysis. Procedia Comput Sci.

[47] Brownlee J (2017). A gentle introduction to the bag-of-words model. Deep Learning for Natural Language Processing.

[48] Zhang Y, Jin R, Zhou Z (2010). Understanding bag-of-words model: a statistical framework. Int J Mach Learn Cyber.

[49] Lei W, Hoi SC, Nenghai Y (2010). Semantics-preserving bag-of-words models and applications. IEEE Trans Image Process.

[50] Singh P Fundamentals of Bag of Words and TF-IDF. Medium.

[51] Havrlant L, Kreinovich V (2017). A simple probabilistic explanation of term frequency-inverse document frequency (tf-idf) heuristic (and variations motivated by this explanation). Int J Gen Syst.

[52] Christian H, Agus MP, Suhartono D (2016). Single document automatic text summarization using term frequency-inverse document frequency (TF-IDF). ComTech.

[53] Peng T, Liu L, Zuo W (2013). PU text classification enhanced by term frequency-inverse document frequency-improved weighting. Concurrency Computat Pract Exper.

[54] Alshamsi A, Bayari R, Salloum S (2020). Sentiment analysis in English texts. Adv Sci Technol Eng Syst J.

[55] Mikolov T, Chen K, Corrado G, Dean J (2013). Efficient estimation of word representations in vector space. arXiv.

[56] Ali Z A simple Word2vec tutorial. Medium.

[57] Ma L, Zhang Y (2015). Using Word2Vec to process big text data. Proceedings of the 2015 IEEE International Conference on Big Data (Big Data).

[58] Lilleberg J, Zhu Y, Zhang Y (2015). Support vector machines and Word2vec for text classification with semantic features. Proceedings of the 2015 IEEE 14th International Conference on Cognitive Informatics & Cognitive Computing (ICCI*CC).

[59] Ma E Understand how to transfer your paragraph to vector by doc2vec. Towards Data Science.

[60] Le Q, Mikolov T (2014). Distributed representations of sentences and documents. Proceedings of the 31st International Conference on Machine Learning.

[61] Chang W, Xu Z, Zhou S, Cao W (2018). Research on detection methods based on Doc2vec abnormal comments. Future Generation Comput Sys.

[62] Karvelis P, Gavrilis D, Georgoulas G, Stylios C (2018). Topic recommendation using Doc2Vec. Proceedings of the 2018 International Joint Conference on Neural Networks (IJCNN).

[63] Bilgin M, Şentürk IF (2017). Sentiment analysis on Twitter data with semi-supervised Doc2Vec. Proceedings of the 2017 International Conference on Computer Science and Engineering (UBMK).

[64] LaValley MP (2008). Logistic regression. Circulation.

[65] Bhargava K, Katarya R (2017). An improved lexicon using logistic regression for sentiment analysis. Proceedings of the 2017 International Conference on Computing and Communication Technologies for Smart Nation (IC3TSN).

[66] Omari MA, Al-Hajj M, Hammami N, Sabra A (2019). Sentiment classifier: logistic regression for Arabic services’ reviews in Lebanon. Proceedings of the 2019 International Conference on Computer and Information Sciences (ICCIS).

[67] Wasi N, Abulaish M (2020). Document-level sentiment analysis through incorporating prior domain knowledge into logistic regression. Proceedings of the 2020 IEEE/WIC/ACM International Joint Conference on Web Intelligence and Intelligent Agent Technology (WI-IAT).

[68] Breiman L (2001). Random forests. Mach Learn.

[69] Karthika P, Murugeswari R, Manoranjithem R (2019). Sentiment analysis of social media network using random forest algorithm. Proceedings of the 2019 IEEE International Conference on Intelligent Techniques in Control, Optimization and Signal Processing (INCOS).

[70] Aufar M, Andreswari R, Pramesti D (2020). Sentiment analysis on YouTube social media using decision tree and random forest algorithm: a case study. Proceedings of the 2020 International Conference on Data Science and Its Applications (ICoDSA).

[71] Singh NK, Tomar DS, Sangaiah AK (2018). Sentiment analysis: a review and comparative analysis over social media. J Ambient Intell Human Comput.

[72] Rustam F, Khalid M, Aslam W, Rupapara V, Mehmood A, Choi GS (2021). A performance comparison of supervised machine learning models for Covid-19 tweets sentiment analysis. PLoS One.

[73] Chen T, Guestrin C (2016). XGBoost: a scalable tree boosting system. Proceedings of the 22nd ACM SIGKDD International Conference on Knowledge Discovery and Data Mining.

[74] Hama Aziz RH, Dimililer N (2021). SentiXGboost: enhanced sentiment analysis in social media posts with ensemble XGBoost classifier. J Chinese Institute Eng.

[75] Hearst M, Dumais S, Osuna E, Platt J, Scholkopf B (1998). Support vector machines. IEEE Intell Syst Their Appl.

[76] Noble WS (2006). What is a support vector machine?. Nat Biotechnol.

[77] Esparza GG, de-Luna A, Zezzatti AO, Hernandez A, Ponce J, Álvarez M, de Jesus Nava J (2018). A sentiment analysis model to analyze students reviews of teacher performance using support vector machines. Distributed Computing and Artificial Intelligence, 14th International Conference.

[78] Shuai Q, Huang Y, Jin L, Pang L (2018). Sentiment analysis on Chinese hotel reviews with Doc2Vec and classifiers. Proceedings of the 2018 IEEE 3rd Advanced Information Technology, Electronic and Automation Control Conference (IAEAC).

[79] Xia H, Yang Y, Pan X, Zhang Z, An W (2019). Sentiment analysis for online reviews using conditional random fields and support vector machines. Electron Commer Res.

[80] Bhuiyan H, Ara J, Bardhan R, Islam MR (2017). Retrieving YouTube video by sentiment analysis on user comment. Proceedings of the 2017 IEEE International Conference on Signal and Image Processing Applications (ICSIPA).

[81] Cunha AA, Costa MC, Pacheco MA (2019). Sentiment analysis of YouTube video comments using deep neural networks. Artificial Intelligence and Soft Computing.

[82] AbdelFattah M, Galal D, Hassan N, Elzanfaly D, Tallent G (2017). A sentiment analysis tool for determining the promotional success of fashion images on Instagram. Int J Interact Mob Technol.

[83] Ortigosa A, Martín JM, Carro RM (2014). Sentiment analysis in Facebook and its application to e-learning. Comput Human Behav.

[84] Mislove A, Lehmann S, Ahn Y-Y, Onnela J-P, Rosenquist J (2011). Understanding the demographics of twitter users. Proceedings of the Fifth International Conference on Weblogs and Social Media.

